# Postoperative Idiopathic Intussusception in an Adult: A Case Report

**DOI:** 10.70352/scrj.cr.26-0549

**Published:** 2026-10-28

**Authors:** Tomokazu Nakagami, Jyoji Yoshizawa, Shinya Kawano, Shohei Honda, Hiroki Yashita, Akihide Sugiyama, Yu Watarai

**Affiliations:** 1 Department of Pediatric Surgery, Showa Medical University Koto-Toyosu Hospital, Tokyo, Japan; 2 Division of Pediatric Surgery, Children's Medical Center, Showa Medical University Northern Yokohama Hospital, Yokohama, Kanagawa, Japan; 3 Department of Pediatric Surgery, Showa Medical University, Tokyo, Japan

**Keywords:** adult, postoperative intussusception, idiopathic intussusception, postoperative intestinal obstruction

## Abstract

**INTRODUCTION:**

Postoperative idiopathic intussusception occurring at a site unrelated to the surgical field is extremely rare in adults. We report a case in which the condition was diagnosed early, thereby avoiding resection of the intussuscepted bowel.

**CASE PRESENTATION:**

The patient was a 56-year-old woman with severe motor and intellectual disabilities who had been diagnosed with cerebral palsy at 11 months of age. She underwent sigmoidopexy for recurrent sigmoid volvulus, but she developed symptoms of intestinal obstruction on POD 2. Abdominal CT revealed findings consistent with intussusception. On POD 3, a 2nd laparotomy revealed an ileocolic intussusception, and manual reduction was performed without resection of the intussuscepted bowel; a sigmoid colectomy was also performed concomitantly. Eight days after the 2nd surgery, adhesive bowel obstruction developed, and a 3rd laparotomy with adhesiolysis was performed the following day. Thereafter, her postoperative course was uneventful, and she was transferred back to her previous facility.

**CONCLUSIONS:**

Postoperative idiopathic intussusception is a well-recognized postoperative condition in children, but it is extremely rare in adults, with only 10 reported cases to date, including the present case. CT is useful for diagnosis. When a target sign or a bowel-within-bowel appearance suggestive of intussusception is observed, early reduction should be considered.

## Abbreviation

POII
: postoperative idiopathic intussusception

## INTRODUCTION

Intussusception is a condition commonly seen in children and is usually idiopathic in pediatric patients. In contrast, adult intussusception is rare and has heterogeneous etiologies. A recent systematic review of adult intussusception reported that 58% of cases were caused by a pathological lead point, 39% were idiopathic, and 25% were associated with malignancy.^1)^

Intussusception occurring shortly after surgery is referred to as postoperative intussusception. However, in children, this term typically refers to idiopathic intussusception, whereas in adults, it often denotes cases with organic causes—such as Meckel’s diverticulum, tumors, surgical anastomoses, adhesions, or ileus tube placement—that can serve as a lead point. In this report, postoperative intussusception without an identifiable organic or pathological lead point is referred to as POII. This designation does not exclude the possible contribution of postoperative dysmotility or postoperative anatomical changes. POII in adults is extremely rare; we report an adult case of POII in which resection of the intussuscepted bowel was avoided after early diagnosis.

## CASE PRESENTATION

The patient was a 56-year-old woman. Due to aerophagia and chronic constipation, which are often seen in patients with severe motor and intellectual disabilities, her entire colon was dilated. Six months prior to surgery, she developed sigmoid volvulus, which was treated by inserting a transanal drain under fluoroscopic guidance.

Colonoscopy performed at the referring hospital reached the terminal ileum, ruled out inflammatory bowel disease, and revealed no organic lesions from the colon to the terminal ileum other than megacolon. Following the examination, she began to experience recurrent volvulus, which was treated each time by inserting a drain. Because she was managed with fasting, she began to lose weight and was referred to our hospital for preventive treatment of volvulus.

Medical history: The patient was diagnosed with cerebral palsy at 11 months of age and has since received care and medical treatment at home. At age 50, due to the advanced age of her caregiver, the patient was admitted to a facility for individuals with severe motor and intellectual disabilities. There was no history of abdominal surgery.

Physical examination revealed a height of 160 cm, a weight of 36 kg, and a BMI of 13.7 kg/m^2^, with an emaciated appearance. The patient required full assistance with ADLs. She was receiving oral nutrition, and her abdomen was generally distended due to aerophagia. An abdominal X-ray taken at admission revealed mild scoliosis, small bowel gas patterns consistent with aerophagia, and a large amount of fecal impaction due to chronic constipation (**Fig. 1**).

**Fig. 1:**
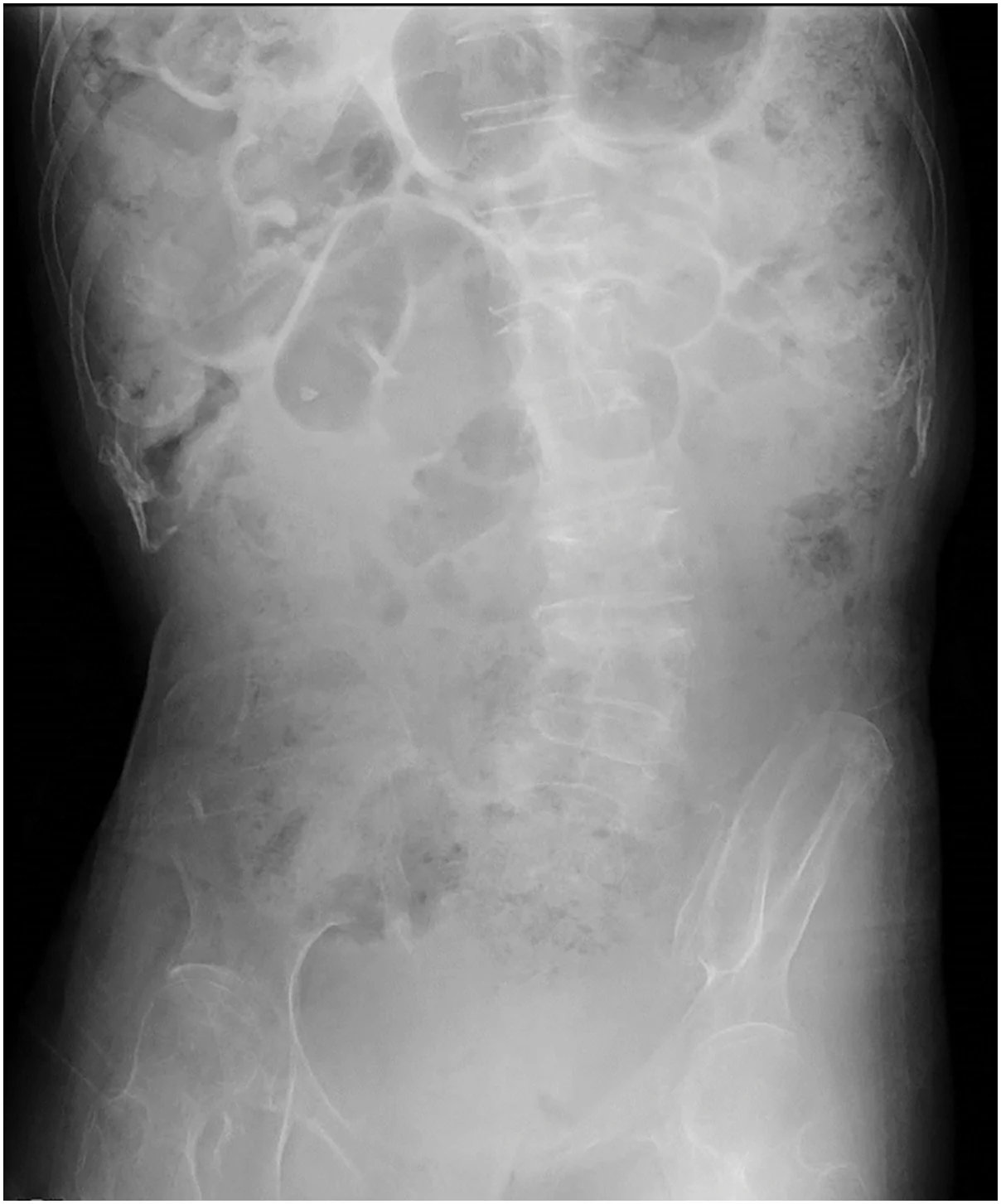
Abdominal X-ray obtained at admission. Gas patterns in the small intestine and fecal impaction are observed.

Following transfer to our hospital, we performed an open sigmoidopexy via a midline laparotomy. The sigmoid colon was markedly dilated and overstretched at a site suspected of previous volvulus. As fixing it at a single point risked recurrence of axial torsion in the remaining segment, we repositioned the sigmoid colon in an inverted U-shape, with the proximal limb on the left and the distal limb on the right, and secured it to both the left and right abdominal walls.

The patient began drinking water on POD 1, but bilious vomiting developed on the POD 2. An abdominal X-ray revealed signs of intestinal obstruction (**Fig. 2**). A contrast-enhanced abdominal CT scan showed dilation of the entire small intestine; the site of obstruction was the terminal ileum, where a target sign was observed (**Figs. 3** and **4**). As her symptoms did not improve, a second laparotomy was performed on the POD 3.

**Fig. 2:**
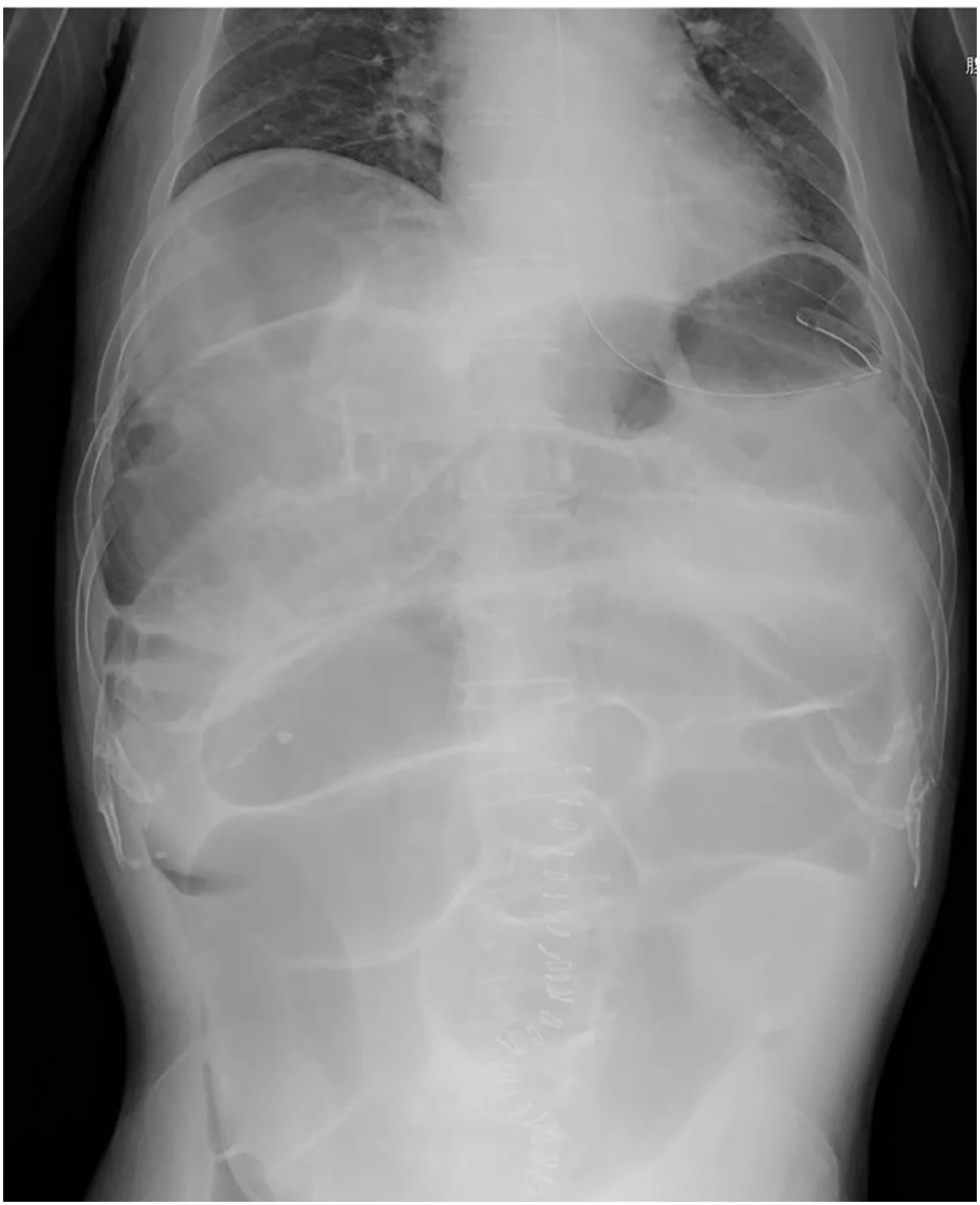
Abdominal X-ray obtained on POD 2. Dilated small intestine and Kerckring folds are observed.

**Fig. 3:**
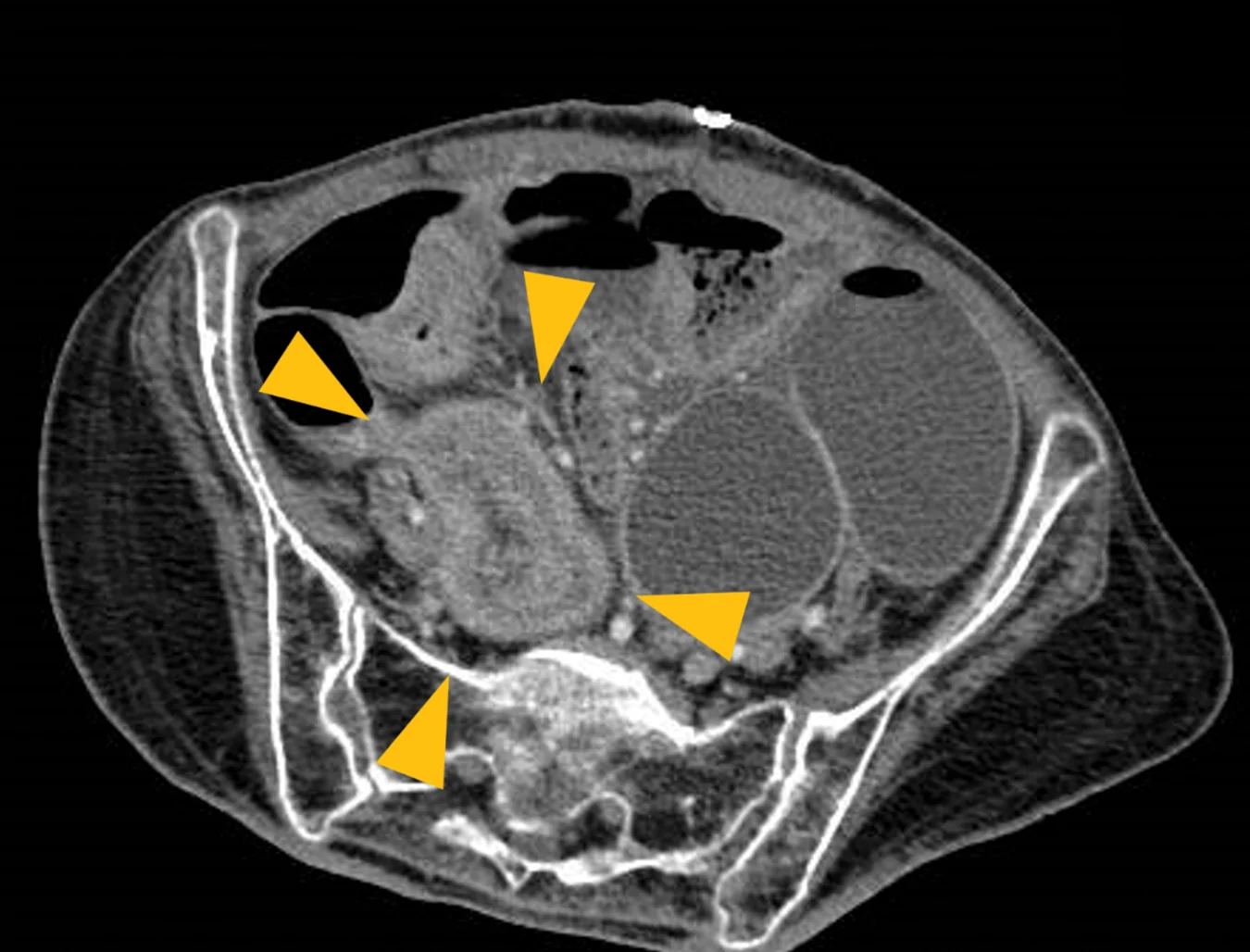
Axial contrast-enhanced abdominal CT image. Yellow arrowheads indicate the target sign.

**Fig. 4:**
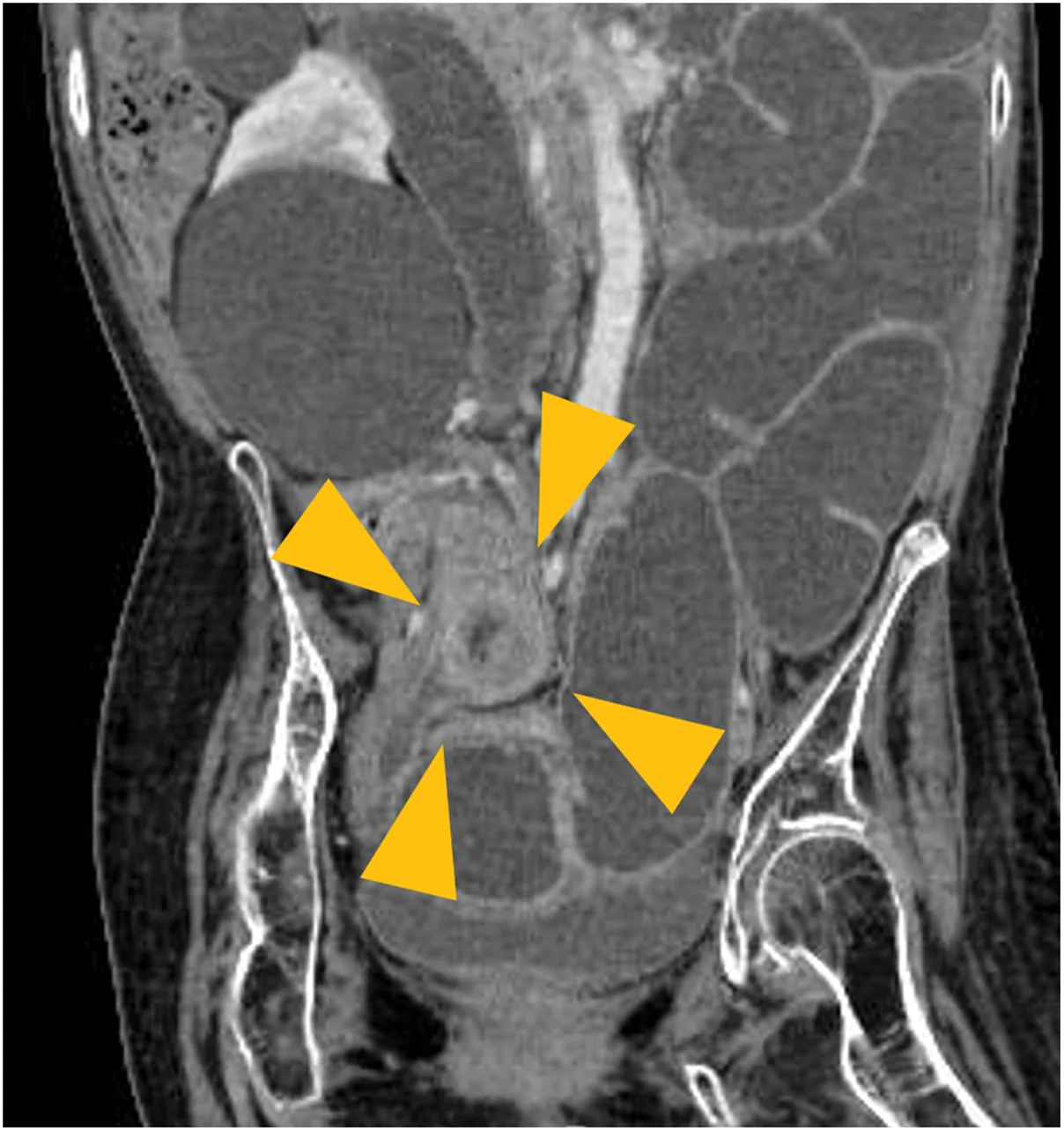
Coronal contrast-enhanced abdominal CT image. Yellow arrowheads indicate the target sign.

At laparotomy, the ileum near the ileocecal valve was found to be intussuscepted in an antegrade direction, with the intussuscepted segment extending beyond the ileocecal valve into the cecum. Based on these findings, the intussusception was classified as ileocolic intussusception (**Fig. 5**). The intussusception was not severe and was easily reduced using the Hutchinson maneuver; no ischemic changes were observed in the intussuscepted bowel. No organic lesion that could serve as a pathological lead point was identified on preoperative CT or during intraoperative inspection and palpation of the intussuscepted bowel. Although no tension or torsion was observed at the site of the sigmoidopexy, part of the fixed sigmoid colon lay over the terminal ileum. Therefore, we considered that the altered position of the sigmoid colon might have contributed to the development of intussusception. We could not rule out the possibility that the markedly dilated sigmoid colon was an extrinsic predisposing factor for the intussusception and posed a risk of recurrence; therefore, sigmoid colectomy was performed despite the patient’s poor nutritional status. Histopathological examination of the resected sigmoid colon showed thickening of the muscularis propria, with no evidence of malignancy, ischemic changes, or other specific histopathological abnormalities, including underlying mucosal lesions. This muscular hypertrophy was considered a secondary adaptive remodeling change resulting from chronic constipation and recurrent sigmoid volvulus.

**Fig. 5:**
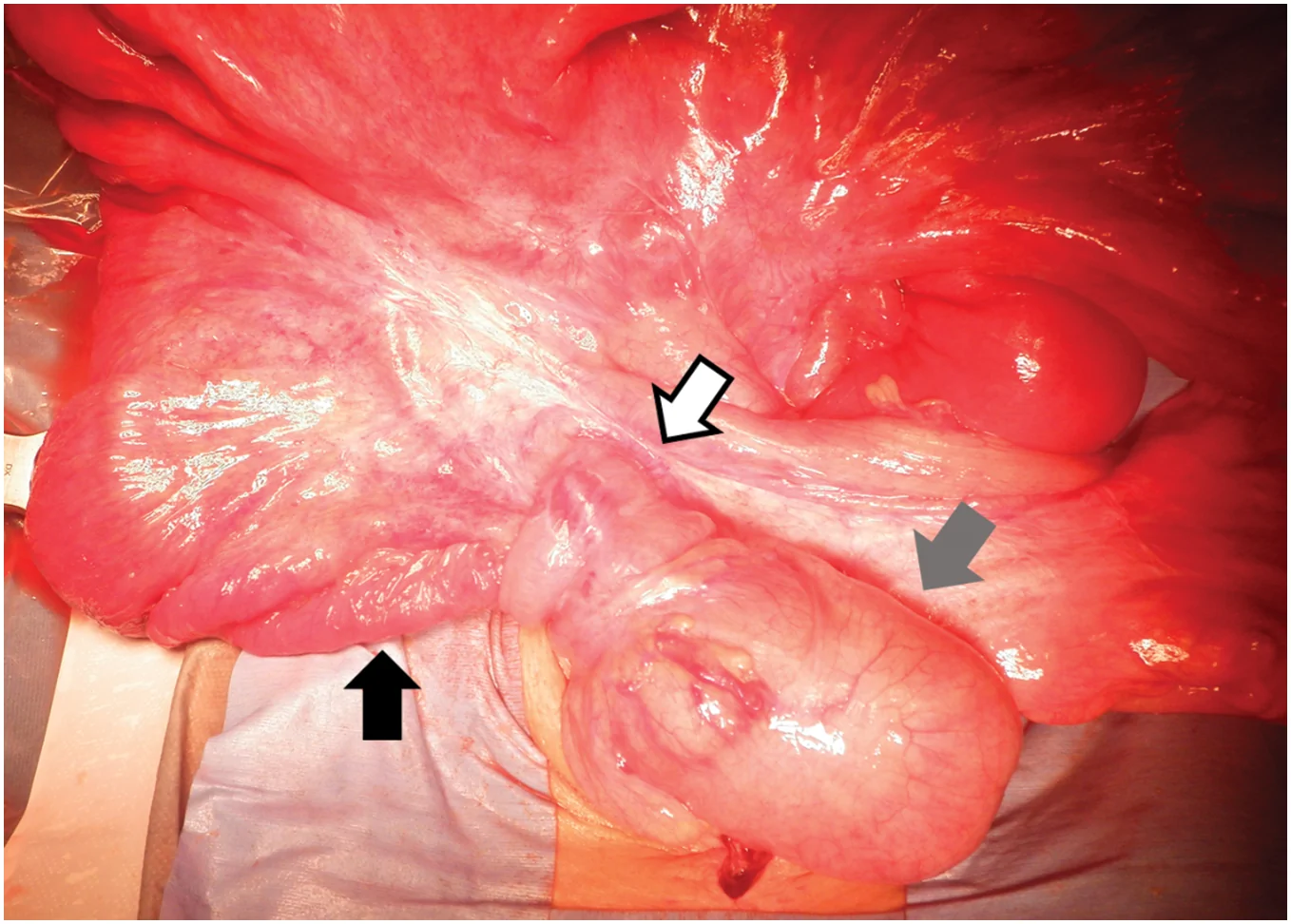
Intraoperative photograph. Black arrow: proximal ileum; white arrow: terminal ileum; gray arrow: cecum. The proximal ileum was intussuscepted into the distal ileum and extended as far as the cecum. This was classified as ileocolic intussusception.

Following reduction of the intussusception, oral fluid intake was permitted on the POD 2, and enteral nutrition was initiated. On the 8th day after the 2nd surgery, symptoms of intestinal obstruction reappeared. A CT scan revealed an obstruction site at the terminal ileum, as in the previous episode, but no target sign was observed. Suspecting adhesive bowel obstruction, a 3rd laparotomy was performed on the 9th day after the 2nd surgery. The terminal ileum, which had been intussuscepted previously, had adhered to the anastomotic site of the sigmoid colon, causing intestinal obstruction. After confirming that the intestinal obstruction had resolved following adhesiolysis, we re-examined the previously intussuscepted bowel but found no organic lesions that could serve as a lead point. The patient’s subsequent course was uneventful, and she was transferred back to her previous facility on the 30th day after the 3rd surgery.

No recurrence of intussusception was observed during 3 years of follow-up.

## DISCUSSION

POII in children has been reported to occur in 0.01%–0.25% of surgeries; although rare, it is a well-recognized condition.^2–4)^ In a review by Yang et al., among reported pediatric POII cases, the preceding operations involved the gastrointestinal system in 51.2%, retroperitoneal tumor surgery in 20.5%, diaphragmatic surgery in 9.4%, and urological surgery in 6.3%.^5)^ It is also known to occur frequently after surgery for neuroblastoma.^6)^

POII in adults is extremely rare. The literature search was completed in May 2026. PubMed was searched using the following search terms: adult AND (“postoperative intussusception” OR “postoperative invagination”). In addition, the reference lists of the identified articles were manually screened to identify additional eligible reports. Adult postoperative cases without an identifiable organic or pathological lead point were included, whereas cases in which tumors, Meckel’s diverticulum, surgical anastomoses, adhesions, or ileus tube placement were identified as the lead point or primary cause were excluded. This search and manual reference-list screening identified 9 previously published cases of POII in adults; including the present case, 10 cases were analyzed (**Table 1**).^7–15)^

**Table 1:** Reported cases of POII in adults

First author	Publication year	Age (years)	Sex	Primary disease	Surgical approach	Initial operation	POD of symptom onset	CT examination	Type of intussusception	Days from symptom onset to intervention	Treatment	Bowel necrosis	Outcome
Kjellström BT^7)^	1991	43	Male	Postoperative ileus	Open surgery	Adhesiolysis	Not reported	Not reported	Jejunojejunal	13	Small bowel resection	No	Alive
Maroju NK^8)^	2004	40	Male	Duodenal perforation	Laparoscopic surgery	Omental patch repair	8	No	Ileocolic	2	Reduction	No	Alive
Zbar AP^9)^	2007	45	Male	Duodenal ulcer	Open surgery	Vagotomy and pyloroplasty	4	No	Jejunojejunal	5	Manual reduction	No	Alive
Jawale A^10)^	2010	32	Female	Colonic tumor	Open surgery	Transverse colectomy	4	No	Jejunojejunal	17	Small bowel resection	Yes	Alive
Harma M^11)^	2011	27	Female	Toxemia of pregnancy	Open surgery	Cesarean section	2	Yes	Ileoileal	3	Manual reduction	No	Alive
Hussain AS^12)^	2014	75	Male	Recurrent rectal cancer	Laparoscopic surgery	Low anterior resection	22	Yes	Ileoileal	Not reported	Small bowel resection	No	Alive
Kim YW^13)^	2016	56	Female	Rectal cancer	Laparoscopic surgery	Low anterior resection	3	Yes	Ileoileal	3	Small bowel resection	Not reported	Alive
Aberra Y^14)^	2017	30	Female	Torsion of an ovarian cyst	Open surgery	Ovarian cystectomy	4	Yes	Ileoileal	8	Manual reduction	No	Alive
Romano M^15)^	2020	42	Male	Rectal cancer	Laparoscopic surgery	Ultralow anterior resection	2	Yes	Jejunojejunal	0	Oral administration of a contrast agent	No	Alive
Present case	—	56	Female	Sigmoid volvulus	Open surgery	Sigmoidopexy	2	Yes	Ileocolic	1	Manual reduction	No	Alive

POII, postoperative idiopathic intussusception

The median time to symptom onset among the 10 cases was 4 days postoperatively. CT was performed in 6 cases, and the mean interval from symptom onset to surgery or intervention was 3 days in cases diagnosed by CT and 8 days in cases not diagnosed by CT. Because the number of cases was small and the clinical contexts were heterogeneous, these findings should be interpreted descriptively. Intestinal necrosis was reported only in cases in which more than 2 weeks had elapsed from symptom onset; however, no firm conclusion can be drawn regarding the relationship between diagnostic timing and necrosis. Three patients underwent bowel resection in cases in which bowel necrosis was either absent or not reported. Two of these 3 patients had undergone surgery for tumors. Therefore, bowel-preserving management may be considered only in selected patients when the bowel is viable and no suspicious lesion is identified.

POII has been attributed to increased postoperative intestinal motility; however, its precise mechanism remains unclear, even in relatively well-described pediatric cases.^3)^ Idiopathic intussusception has also been reported after trauma, even in the absence of surgery.^16,17)^ Komadina et al. suggested that intussusception may be caused by local spasm of a bowel segment and abnormal peristalsis.^18)^ At the initial operation in the present case, the sigmoid volvulus had already been reduced. Dilatation was observed only in the sigmoid colon, and there was little retention of intraluminal contents; therefore, decompression was not performed. The subsequent intussusception occurred in the terminal ileum, a bowel segment that had not been directly manipulated during the initial operation. These findings suggest that postoperative intestinal dysmotility may have been involved. Although the intussusception was classified as idiopathic because no identifiable organic lesion serving as a lead point was found, the preceding sigmoidopexy and the altered positional relationship between the fixed sigmoid colon and the terminal ileum may have acted as extrinsic predisposing factors. Given the patient’s history of cerebral palsy, autonomic dysfunction may also have influenced intestinal motility; however, this possibility remains speculative, and no direct causal relationship can be established.

Considering the time from symptom onset to treatment in cases of POII, early CT evaluation is useful. In adults, when intestinal obstruction is present, US may be difficult to interpret due to intestinal gas; however, CT is the most useful imaging modality for diagnosing intussusception, with a recent systematic review reporting a correct preoperative diagnosis in 82% of cases based on 17 studies.^1)^ In the present case, knowledge from pediatric surgery regarding POII prompted a CT-based diagnosis, and early surgery enabled bowel preservation. For early postoperative intestinal obstruction, a CT scan should be performed to determine whether the obstruction is mechanical or paralytic. When CT demonstrates a target sign or a bowel-within-bowel appearance suggestive of intussusception at the site of obstruction, early reduction should be considered. When the underlying disease is a malignant tumor, resection should be considered even when bowel perfusion is preserved.

## CONCLUSIONS

POII is a well-recognized postoperative condition in children, but it is extremely rare in adults, with only 10 cases reported to date, including the present case. CT is useful for diagnosis, and when CT demonstrates a target sign or a bowel-within-bowel appearance suggestive of intussusception, prompt reduction should be considered.

## DECLARATIONS

### Availability of data and materials

The datasets supporting the conclusions of this article are included within the article.
